# Exploratory study of the implications of research on the use of smart connected devices for prevention: a scoping review

**DOI:** 10.1186/s12889-016-3225-4

**Published:** 2016-07-11

**Authors:** Audrey Petit, Linda Cambon

**Affiliations:** Chaire de recherche en prévention des cancers, UMR6051, CRAPE, EHESP, Rennes, France; EHESP, 20 Avenue George Sand, 93210 Saint-Denis, France

**Keywords:** Smart devices, ehealth, Quantified self, Prevention, Health care relationship

## Abstract

**Background:**

Smart devices and mobile applications are now an integral part of all aspects of everyday life. They are particularly numerous in the field of health, contributing to the movement called ehealth. What is the potential role of these devices as prevention supports? The purpose of this article is to provide an exploratory analysis of the use, efficacy and contribution to conventional prevention strategies.

**Methods:**

To address this issue, we conducted a scoping-review on the basis of 105 publications from the fields of medicine and human sciences.

**Results:**

Three dimensions of the use of smart devices in the field of health were identified: 1/a quantification tool allowing the users to measure their activities; 2/a tool of self-positioning in the community; 3/an interface between the medical world and the population, modifying the hierarchy of knowledge. However, few published studies have investigated the determinants of the efficacy of these devices and their impact on individual behaviours and professional health practices.

**Conclusion:**

Based on the hypothesis of possible integration of these devices in prevention policies, it would be interesting to investigate two research issues: how and under what psycho-socio-environmental conditions can smart devices contribute to the adoption of positive health behaviours? To what degree does the use of smart devices modify the health care professional-patient relationship? Finding answers to these questions could help to define the real place of these devices in prevention strategies by determining their complementarity with respect to other prevention strategies, and the conditions of their efficacy on behaviours and inequalities.

## Background

Smart devices and/or communicating mobile devices [1, 2] are internet-connected devices that provide the user with information and an interaction with the environment, specifically linked to a system of identification, sensing and transmission of data (outside temperature, heart rate, etc.) to an application present on an interface (for example, a smartphone) [3]. A considerable number of these devices and applications, for both the general public and various business sectors, have been released onto the market over recent years [4] and are now an integral part of all aspects of everyday life. For example, in 2013, there were about 9 billion smart devices in the world, i.e. 1.25 smart devices per person [5].

A large proportion of these innovations concern the field of health. These devices, ranging from internet-connected tablet containers to connected wristbands or heart rate monitors and medical alert necklaces for the elderly, are an integral part of the “ehealth” movement [6–8] that was initiated at the end of the 1990s. This movement is defined as the use of emerging mobile communications in public health [5, 9–12] designed to change health behaviours and health care. It has been defined as an ally of medicine and biomedical research [13]. About 100,000 applications are now available in the health sector, 70 % of which are related to the well-being segment, concerning almost 5 million people in France [14]. The development of these devices in the health care sector, governed in France by Article L.6316 of the French public health code, is also intensive and diversified: rating scale for chemotherapy sessions and associated adverse effects, textual interpretation of arterial blood gases, management of diabetic patients by a web-based telemonitoring platform [15], teleradiology-based management of neuroradiology emergencies [16], telemedicine applied to muscle rehabilitation [17], telemonitoring in patients with heart failure [18], etc.

Consequently, in the field of health, also marked by increasing life expectancy, an increasing number of chronic diseases and the growth of outpatient management [19, 20], these devices will inevitably occupy an increasingly important place alongside conventional curative and preventive health policies and management. However, few data are available, especially in the field of prevention. What is the real value of these devices as a support for prevention behaviours [21, 22]? What questions are currently raised in the literature concerning their use, their efficacy or their contribution to conventional strategies. This article is designed to address these various issues based on a review of the literature.

## Method

In order to address these issues, we conducted a scoping review [23], which can be defined as “exploratory projects that systematically map the literature available on a topic, identifying key concepts, theories, sources of evidence and gaps in the research. They are often preliminary to full syntheses, undertaken when feasibility is a concern - either because the potentially relevant literature is thought to be especially vast and diverse.... or there is a suspicion that not enough literature exists” [24]. We applied the PRISMA guidelines [25] (relevant items: eligibility criteria, information sources, search, study selection).

We performed a literature search using the following key words: prevention OR education AND e-health OR m-health OR health education AND coaching OR prevention on the Web of Science database. This database was selected because it is a multidisciplinary database that includes the best scientific journals, including in the field of human sciences. We searched for all original and methodological articles indexed between 2000 and 1st December 2015, in English or in French, and selected relevant articles on the basis of their abstracts according to the following criteria: articles concerning the use of smart devices and/or health applications, articles in the field of prevention (in the health system and in other settings), articles concerning modalities of use and/or impact on users, articles on general public interventions. Articles on the curative use of smart devices were excluded. The articles identified were selected by double reading using Covidence software [26]. Certain articles not meeting our selection criteria and not initially selected, but cited in selected articles and likely to be interesting to assess the scope of the subject, were then identified and were added to the selection (doctorate theses, dissertations, didactic articles, methodological articles, scientific articles including human sciences but not in the field of prevention).

We then analysed the selected articles, on the basis of the complete text, according to two questions: What are the objectives of using smart devices in prevention? What questions are raised by the use of smart devices in relation to the conventional prevention strategy? Finally, several other articles, listed in the references of the selected articles, were also progressively included in the analysis, especially human science articles or articles on ehealth, but not concerning the field of prevention.

## Results

**Fig. 1:**
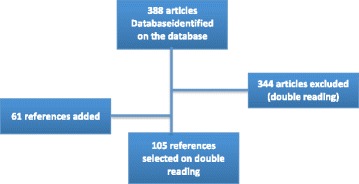
Number of articles selected

Of the 388 articles selected by the search algorithm, 44 were selected on the basis of the inclusion criteria. Excluded articles were mainly excluded because they concerned information technology techniques or curative use (especially telemedicine). Another 61 articles were added to this first selection. A total of 105 publications were finally included in the analysis (see Table 2 in the Appendix) (Fig. 1).

The articles were analysed and classified according to two questions. In the first question concerning the purpose of using a smart device, three dimensions were identified: for the purposes of quantification, for the purposes of socialization, for modification of the doctor-patient relationship. This classification is presented in Table 1 below.

**Table 1:** List of articles by field of analysis

	Purposes of smart device use			Modification of prevention strategies
	(87 references)			
	Quantified-self	Socialisation	Doctor-patient relationship	
	(24 references)	(10 references)	(52 references)	(23 references)
Article No.	1; 2; 7; 8	5		
	10		13; 14; 15; 16; 17; 18; 19	14
	27; 28; 29;		20; 22; 28	
	30; 31; 32; 33; 34; 35; 36; 37; 38; 39	32; 35; 37; 39;		
	44	40; 41; 42; 43; 46	45; 46; 47; 48; 49;	46
			50; 52; 53; 54; 55; 56; 57; 58; 59	52; 53
			60; 61; 62; 63; 64; 65; 66; 67; 68; 69	
			70; 71; 72; 73; 74; 75; 76; 77; 78; 79	72
	88; 89		80; 81; 82; 83; 84; 85; 86; 87	88; 89
	90; 91; 92			90; 91; 92; 93; 94; 95; 96; 97; 98; 99
				100; 101; 102; 103; 104; 105

### Purposes of smart device use

Three dimensions of the use of smart devices in prevention were identified.

#### Quantified self devices: objective monitoring of health and health behaviours

The first dimension considered in the literature concerns quantification of health-related activities or constants [27, 28], corresponding to the socio-technological “quantified self” movement, also called self-quantifying, self-surveillance, or self-tracking, developed in the United States at the initiative of two *Wired* magazine journalists, Gary Wolf and Kevin Kelly. This movement has grown considerably over the last 10 years, with the publication of a number of books devoted to its philosophy, quantified self guides [7, 29, 30] and the creation of a quantified self collective [30]. This movement promotes self-knowledge based on figures provided by connected body sensors on scales, blood pressure monitors or pedometers that send information to a smartphone (m.health [31]). One of the aims of this movement is to quantify all activities or all subjects [32] by means of algorithms [33], even subjective variables such as pain or mood [28].

More specifically, especially in the field of prevention, these devices are designed to collect, measure and compare various biological, physical, behavioural and environmental parameters concerning lifestyle activities such as sleeping, eating and physical exercise, in order to improve well-being and maintain or improve the subject’s state of health [34], but also to measure the subject’s consumptions (for example smoking, alcohol, calories) or activities (work time, leisure activities, physical exercise, etc.). Some of these data (e.g.: blood pressure, pulse) were conventionally recorded and analysed in the specific setting of the doctor-patient relationship in the context of a specific risk [35]. The quantified self therefore modifies the frontiers between the fields of well-being, health and health care, which now constitute a continuum between normal and pathological rather than a break-point [10, 36] The objective for users is to collect data in order to acquire knowledge about themselves and their health in the form of graphic representations reflecting the time-course of selected variables [28]. Self-quantification induces a perception of the body that is modelled in an essentially technical relationship determined by quantitative data.

Self-quantification is therefore a way for individuals to objectively visualize their behaviours [37], as part of a strategy of self-knowledge and self-construction [38], although these strategies are not always maintained in the long term [32, 35, 39].

#### Smart devices as a means of socialization: a source of social valorization

The second dimension studied in the literature concerns sharing of the data collected and analysed by users of smart devices. Users of these technologies generally belong to internet-connected communities with a double objective of valorization of their efforts and encouraging reassurance according to various configurations. These devices are therefore part of the social interaction economy [40] that highlights the fact that behavioural dynamics are linked to the dynamics of social relationships, whose existence depends on the effects of influence exerted within social groups.

As an example, *Lab Orange* researchers [32] have defined three types of modalities of use of these measurements. The first consists of surveillance, corresponding to measurement of a risk, in which the concept of threshold plays a central role and is usually defined by external, often medical, norms. This is the case, for example, of body mass index (BMI). This modality does not focus on action, but on self-surveillance. Consequently, the results of this self-surveillance can sometimes be a source of anxiety and may therefore not lead to data sharing. According to this modality, advice is then generally shared on social networks according to a logic of mutual aid and support. The second modality concerns what is known as routinization or regularity, which is designed to replace a bad habit by a more favourable health behaviour, for example smoking cessation or adoption of lifestyle and dietary measures. In contrast with the first modality, this modality comprises an action or a change in which the central element is regularity driven by motivation. In this case, publication of the individual’s measurements on social networks is designed to arouse encouragement, but the subject may also prefer to avoid other peoples’ opinions. Finally, the third modality refers to performance and the various measurements become self-determined objectives. The objective of this modality is to enhance motivation and improve performance. Social networking allows both sharing of experiences as well as competition and the norms derived from the challenge.

In all three cases, these sharing practices constitute tools of technological mediatization and social mediation [37] allowing renewed forms of self-exposure [41] or self-narration [42]. However, networking does not appear to constitute “a standardization of private activities. Although they are driven by the promoters of these tools and the supporters of the quantified self, discussions between users are rare and alignments of practices between the various users does not appear to constitute a dominant expectation” [32]. Moreover, these measuring practices tend to decline with time [32, 35], as more than one-third of users stop using their smart device in less than 6 months [5] due to a phenomenon described by the law of attrition [39]. Sharing of measurements recorded by smart devices corresponds to a socialization practice, in which the measurement provides an opportunity to communicate according to new codes [43, 44].

#### Smart devices in the health care relationship: a mediator of participative medicine

The third dimension identified by this review concerns the medical setting and involves transformations of health care practices related to the emergence and the potential place of these devices in preventive medicine practices [28, 45].

The literature on this aspect emphasizes the empowerment potential [46, 47] of patients with respect to health care professionals resulting from the use of these devices, allowing them to become active partners of their own health [48]. The use of smart devices in the preventive or curative health care relationship introduces a form of “media medicine” [49] or apomediation [46] meaning remote mediation between the patient and his/her body detached from the doctor, which results in a new doctor/patient relationship articulated around scientific and lay knowledge [19] leading to the emergence of new health care models [28, 50]. The patient becomes patient-expert and the doctor accompanies the patient in his/her life trajectory [14], by replacing a repair strategy by a lifelong support strategy [51] of an empowered and networking patient [52–54], corresponding to an ascending approach to medicine, which could result in a knowledge competition between health care professionals and their patients [55]. This competition is increasing in parallel with the growth of a large on-line community [13, 28, 56–61] and information sharing, concerning both disease and healthy lifestyles, which redefine the hierarchy of knowledge [19]. In France, for example, more than one half of the population and 61 % of subjects with a chronic disease search the internet for health-related information [50]. Patient communities describe diseases in terms of personal experience [52, 62] by means of peer training, information sharing and networking to more effectively manage their health [52, 53]. These patient networks can constitute a new partner in the health care ecosystem [28]. In contrast, few studies have investigated how patients use this information in a context in which the quality of on-line information is not always reliable [57, 63]. Furthermore, sharing of personal information, previously exclusively confided to doctors, raises ethical issues concerning their use and their confidentiality [64, 65]. Studies examining health care professionals’ perception and integration of these devices in preventive medicine practices also highlight the obstacles to their use [66–71] and the need to train both users [72, 73] and health care professionals [74–80] or even the creation of new medical specialties at the interface between information technology and medicine [81, 82]. Finally, these technologies can also impact on relationships between professionals themselves [82] and consequently on the distribution of tasks concerning the patient and his/her care pathway [82–84], leading to more mutualization and less autonomy of professionals.

The use of these devices in the preventive or curative health care relationship is accompanied by new alliances and conflicts between health care professionals and a different sharing of decisions between patients and professionals [52, 55, 85–87]. These changing relations impact on both the nature of the therapeutic alliance, redefining the balance of knowledge between the patient and the health care professional, and the modalities of elaboration of the therapeutic alliance, redefining sharing of skills of the various professionals participating in the patient’s care pathway.

These three modalities of use suggest that these devices could possibly contribute to new prevention models.

### Smart devices as a support for behaviour change: marker of a new prevention model

In the light of this review of the literature, these three dimensions of the use of smart devices raise the question of their possible effects on health-related behaviour. The underlying hypothesis is that objective demonstration of behaviours (quantified self) may contribute to transformation of the subject’s relationship to his/her body and health by adoption and consequently normalization of certain behaviours [88] that could be targeted in prevention policies. Other authors have emphasized the effect of these devices but on the basis of other factors. These devices would therefore contribute to behaviour change [72] and the emergence of a new representation of the body and health by promoting empowerment, which cannot be achieved by the biomedicine model [89]. Empowerment is “an individual’s capacity to take decisions when faced with a specific situation or problem, either alone or by group participation, in order to adapt to this situation and take control of their personal life” [14]. In other words, by means of objective measurement of their health and behaviour, individuals would be more able to make more favourable adaptive choices. Several studies [90–92] have corroborated this hypothesis in the clinical practice setting, by showing that the “patient’s implication in management of his or her treatment has beneficial effects […]. It improves treatment adherence in many diseases and doctors are currently trying to develop tools that can enhance this implication” [52]. The efficacy of ehealth, in the broad sense of the term, has therefore been demonstrated in many fields such as overweight and obesity [93, 94], HIV [95–97], cancer [98–100] and diabetes [92, 101]. Nevertheless, few studies have demonstrated the efficacy and use of these smart devices with respect to conventional strategies. The question of their universal accessibility and consequently the social inequalities that can be induced by the use of these devices [102, 103], also needs to be investigated, as there is a risk that the digital divide [53, 104] may further accentuate the health divide between users with access to this technology and those without access to this technology [46].

The use of these devices as a support for health behaviour changes, and therefore as a specific prevention tool, needs to be further investigated both in terms of the way in which these devices act (empowerment versus normalization), and their efficacy and contribution to the problems of social and regional health inequalities.

## Discussion

Although not as comprehensive as a systematic review of the literature, this scoping review provides a fairly precise overview of the research issues present in the literature concerning the use of smart devices in prevention strategies, either outside of the medical field (health and well-being) or in the context of clinical prevention practices (health and prevention of diseases or their complications). We have limited our research to the field of prevention and we have excluded the very abundant literature on curative aspects and have also included publications derived from the fields of human and social sciences. We consequently observed that a large number of articles were excluded and many articles not meeting our inclusion criteria also had to be added (*n* = 61). These screening failures could be explained by two hypotheses. Firstly, our search algorithm was too broad: in particular, the word “coaching” refers to support, essentially therapeutic support, but not necessarily associated with a smart device or an application. The use of this search term selected a large number of irrelevant articles. The second hypothesis concerns the salience of this innovative subject, especially in the so-called grey literature (not referenced in scientific databases) and in fields not related to health.

Three main dimensions were identified. Each dimension situates the smart device in the context of a specific objective and a specific use. The first dimension positions the device as a tool for quantification of activity, allowing users to measure their activities, assess their progress and project themselves towards a target. It consequently constitutes a self-construction tool providing an objective measure of self-control, assuming that the self can be defined by these variables. The second dimension concerns self-positioning in the community. As a vector of collective socialization, the device provides an opportunity to seek advice and encouragements. The third dimension is that of a mediator between an environment considered up until now to be a source of knowledge, the medical environment, and the population. This mediation breaks down the barriers of knowledge, redefining the relationship between patients and health care professionals and between professionals concerning curative or preventive management, which is consequently transformed in terms of its scientific basis and its methods. In view of these elements, smart devices can be considered to be tools that could be integrated into the conventional prevention arsenal, and therefore subject to the same fundamental questions: what is the final objective (empowerment versus normalization)? And what is the impact on social and regional health inequalities [105]?

In reality, these findings highlight a blind spot in the literature: explanation of the mechanisms of efficacy of these devices and their impact on health practices and professional practices, as few studies have investigated the mechanisms mobilized by the use of these devices in favour of health behaviours. In fact, beyond the question of the quality and reliability of the data and algorithms integrated into these devices, their objectives and the scope of their use in prevention need to be precisely defined: What are the psychosocial mechanisms underlying the use of these devices for the purposes of health? What are the objectives of these devices: to monitor, improve performance, accompany behaviour changes, develop empowerment, etc.? In what types of populations are they relevant (age, gender, socioeconomic category, medical history)? In what way do they compensate or complete conventional strategies? What socio-environmental factors potentiate or limit the effects of these devices on behaviour change? The data derived from the literature also fail to provide any details on the absolute efficacy or the efficacy according to social gradient of these devices in the field of prevention nor the conditions of this efficacy.

Finally, very few data are available in the literature to explain the transformations of practices induced by the use of smart devices in the health care relationship and the impact of this transformation on the health system and its capacity to provide an egalitarian response to the population’s needs, as it is unclear from this scoping review whether these changes apply to all fields of prevention and health care and all types of patients. How do health care professionals adapt to these new practices? More broadly, how is the health system preparing for this transformation and what changes will be required in the training of health care professionals?

## Conclusion

To conclude, this scoping review identified three different dimensions concerning the use of smart devices in prevention. Based on the hypothesis of integration of these devices into prevention policies, this review emphasizes the importance of investigating two questions that have been poorly studied to date, although they represent a real research challenge in this field: how and under what psycho-socio-environmental conditions can ehealth smart devices contribute to the adoption of positive health behaviour? To what degree and how does the use of smart devices positively or negatively modify the doctor-patient relationship?

Finding answers to these questions could help to define and confirm the real place of these devices in prevention strategies by clearly demonstrating their added value and complementarity with respect to other prevention strategies, and by defining the conditions of their efficacy on behaviours, especially by taking into account the question of social and regional inequalities of access to health care.

## Appendix

**Table 2:** List of articles selected

No.	Year	Author	Language	Type	Selection	Criteria
1	2013	Benferhat	French	Doctorate thesis	Addition	Smart devices In the health system Quantified-self
2	2012	Swan	English	Original article	Database	Health applications Non-health setting Quantified-self
3	2014	Lendrevie	French	Book	Addition	Health applications Non-health setting
4	2014	Bellanger-Trely	French	Vade Mecum	Addition	Smart devices Health applications In the health system and other settings
5	2015	IREPS Bretagne	French	Grey literature	Addition	Smart devices Health applications Modalities of use In the health system and other settings
6	2011	Dupagne	French	Original article	Database	Health applications In the health system
7	2014	Robin	French	Book	Addition	Health applications Modalities of use In the health system
8	2012	Wiederhold	English	Original article	Database	Smart devices Modalities of use In the health system
9	2014	CATEL	French	Guidelines	Addition	Health applications In the health system
10	2015	CNOM	French	Guidelines	Addition	Health applications Modalities of use In the health system
11	2001	Eysenbach	English	Original article	Database	Health applications Modalities of use In the health system
12	2011	Garel	French	Book	Addition	Health applications Modalities of use In the health system
13	2008	Eysenbach	English	Original article	Database	Health applications Impact on users In the health system
14	2014	Salmon	French	Original article	Database	Impact on users Prevention strategies In the health system and other settings
15	2013	Benhamou	French	Original article	Database	Health applications Impact on users In the health system
16	2008	Hazebroucq	French	Original article	Database	Health applications Impact on users In the health system
17	2008	Avraam	English	Original article	Database	Health applications Impact on users In the health system
18	2012	Bignolas	French	Original article	Addition	Health applications Impact on users In the health system
19	2014	Dubey	French	Original article	Database	Health applications Impact on users In the health system
20	2006	Giustini	English	Original article	Database	Health applications Impact on users In the health system
21	2014	Merloz	French	Original article	Database	Health applications In the health system
22	2014	Vial	French	Original article	Addition	Health applications Impact on users In the health system
23	2010	Levac	English	Methodological article	Database	--
24	2010	IRSC	French	Grey literature	Addition	--
25	2008	Mother	English	Methodological article	Addition	--
26	2014	Babineau	English	Methodological article	Addition	--
27	2014	Beauchet	English	Original article	Database	Smart devices Modalities of use In the health system
28	2009	Swan	English	Original article	Database	Smart devices Health applications Modalities of use Impact on users In the health system and other settings
29	2008	Cotteret	French	Book	Addition	Smart devices Modalities of use
30	2012	Gadenne	French	Book	Addition	Smart devices Modalities of use Non-health setting
31	2015	Ahmadvand	English	Original article	Database	Smart devices Modalities of use In the health system and other settings
32	2013	Pharabond	French	Original article	Database	Smart devices Modalities of use Non-health setting
33	1990	Laguna	English	Original article	Addition	Smart devices Modalities of use In the health system
34	2008	Reiter	English	Original article	Database	Smart devices Modalities of use Non-health setting
35	2014	CNIL	French	Grey literature	Addition	Smart devices Modalities of use Non-health setting
36	1966	Canguilhem	French	Book	Addition	Modalities of use
37	2013	Arruabarrena	French	Original article	Addition	Smart devices Modalities of use Non-health setting
38	2012	Mondoux	French	Original article	Addition	Health applications Modalities of use
39	2005	Eysenbach	English		Database	Modalities of use Non-health setting
40	1993	Manski	English	Original article	Addition	Modalities of use
41	2010	Granjon	French	Original article	Addition	Modalities of use
42	2006	Cardon	French	Original article	Addition	Modalities of use
43	2009	Aguiton	English	Original article	Addition	Modalities of use
44	2014	Caldwell	English	Original article	Database	Modalities of use
45	2013	Delmotte	French	Original article	Addition	Health applications Impact on users In the health system
46	2011	Casilli	French	Original article	Addition	Modalities of use Prevention strategies
47	2008	Van Uden-Kraan	English	Original article	Addition	Impact on users In the health system and other settings
48	1999	Charles	English	Original article	Addition	Impact on users In the health system
49	2015	Vallancien	French	Book	Addition	Impact on users In the health system
50	2015	Wernette	French	Original article	Addition	Impact on users
51	2014	Caniart	French	Original article	Addition	Health applications In the health system and other settings
52	2010	Jouet	French	Review	Addition	Impact on users Prevention strategies
53	2015	Brouard	French	Original article	Database	Impact on users Prevention strategies
54	2008	Frost	English	Original article	Addition	Impact on users In the health system and other settings
55	2011	Laubie	French	Original article	Database	Impact on users In the health system and other settings
56	2009	Akrich	French	Original article	Addition	Impact on users Non-health settings
57	2008	Mayoh	English	Original article	Database	Impact on users In the health system and other settings
58	2015	Valdez	English	Original article	Addition	Impact on users In the health system and other settings
59	2015	Magnezi	English	Original article	Addition	Impact on users In the health system and other settings
60	2014	Magnezi	English	Original article	Addition	Impact on users In the health system and other settings
61	2013	Medina	English	Original article	Addition	Impact on users In the health system and other settings
62	2014	Delory-Momberger	French	Original article	Addition	Impact on users In the health system and other settings
63	2008	Mitchell	English	Original article	Database	Impact on users In the health system
64	2014	Béranger	French	Original article	Database	Impact on users In the health system
65	2009	Lucas	French	Original article	Database	Impact on users In the health system
66	2012	Gagnon	English	Methodological article	Database	Impact on users In the health system
67	2012	Gund	English	Original article	Database	Impact on users In the health system
68	2008	Ward	English	Methodological article	Database	Impact on users In the health system
69	2012	Dünnebeil	English	Original article	Database	Impact on users In the health system
70	2009	Eley	English	Original article	Database	Impact on users In the health system
71	2005	Richards	English	Original article	Database	Impact on users In the health system
72	2010	Sandrin-Berthon	French	Book	Addition	Prevention strategies Impact on users In the health system
73	2005	Eymard	French	Original article	Addition	Impact on users In the health system
74	2002	Gros	French	Rapport	Addition	Impact on users In the health system
75	2013	Dattakumar	English	Original article	Addition	Impact on users In the health system
76	2013	Lapao	English	Original article	Addition	Impact on users In the health system
77	2012	Bygholm	English	Original article	Addition	Impact on users In the health system
78	2011	Stellefson	English	Methodological article	Addition	Impact on users In the health system
79	2009	Clark	English	Original article	Addition	Impact on users In the health system
80	2008	Zvarova	English	Original article	Addition	Impact on users In the health system
81	2003	Moulin	French	Original article	Addition	Impact on users In the health system
82	2013	Mathieu-Fritz	French	Original article	Addition	Impact on users In the health system
83	2011	Esterle	French	Original article	Addition	Impact on users In the health system
84	1992	Strauss	French	Book	Addition	Impact on users
85	2012	Andrieu	French	Book	Addition	Impact on users In the health system
86	2009	Silber	French	Original article	Addition	Impact on users In the health system and other settings
87	2009	Silber	French	Original article	Addition	Impact on users In the health system
88	2014	Martin	French	Dissertation	Addition	Health applications Impact on users Prevention strategies Non-health setting
89	2015	Thornquist	English	Original article	Database	Health applications Impact on users Prevention strategies Non-health setting
90	2014	Shull	English	Methodological article	Database	Health applications Impact on users Prevention strategies In the health system and other settings
91	2015	Van den Bulck	English	Original article	Database	Health applications Impact on users Prevention strategies Non-health setting
92	2015	Goyal	English	Original article	Database	Health applications Impact on users Prevention strategies Non-health setting
93	2015	Hutchesson	English	Methodological article	Database	Health applications Prevention strategies In the health system
94	2013	Tate	English	Original article	Database	Health applications Prevention strategies In the health system
95	2014	Odeny	English	Original article	Database	Health applications Prevention strategies In the health system
96	2015	Muessig	English	Original article	Addition	Health applications Prevention strategies In the health system
97	2012	Noar	English	Original article	Database	Health applications Prevention strategies In the health system
98	2013	Sanchez	English	Methodological article	Database	Health applications Prevention strategies In the health system
99	2013	Elliot	English	Original article	Database	Health applications Prevention strategies In the health system
100	2015	Davis	English	Original article	Database	Health applications Prevention strategies In the health system
101	2012	Pacaud	English	Original article	Addition	Health applications Prevention strategies In the health system
102	2014	Steinberg	English	Original article	Database	Health applications Prevention strategies In the health system
103	2009	Atkinson	English	Original article	Addition	Health applications Prevention strategies In the health system
104	2011	Granjon	French	Original article	Addition	Prevention strategies In the health system and other settings
105	2015	Zhang	English	Original article	Addition	Prevention strategies In the health system
